# Lifestyle and clinical factors associated with elevated C-reactive protein among newly diagnosed Type 2 diabetes mellitus patients: a cross-sectional study from the nationwide DD2 cohort

**DOI:** 10.1186/1472-6823-14-74

**Published:** 2014-08-28

**Authors:** Elisabeth Svensson, Anil Mor, Jørgen Rungby, Klara Berencsi, Jens Steen Nielsen, Jacob V Stidsen, Søren Friborg, Ivan Brandslund, Jens Sandahl Christiansen, Henning Beck-Nielsen, Henrik Toft Sørensen, Reimar W Thomsen

**Affiliations:** 1 Department of Clinical Epidemiology, Institute of Clinical Medicine, Aarhus University Hospital, Aarhus, Denmark; 2 Department of Pharmacology, Institute of Clinical Medicine, University of Aarhus, Aarhus, Denmark; 3 Diabetes Research Centre, Department of Endocrinology, Odense University Hospital, Odense, Denmark; 4 Department of Endocrinology M, Odense University Hospital, Odense, Denmark; 5 Department of Biochemistry, Lillebaelt Hospital Vejle, Vejle, Denmark; 6 Department of Internal Medicine and Endocrinology, Institute of Clinical Medicine Aarhus University Hospital, Aarhus, Denmark

**Keywords:** C-reactive protein, Lifestyle factors, Obesity, Physical activity

## Abstract

**Background:**

We aimed to examine the prevalence of and modifiable factors associated with elevated C-reactive Protein (CRP), a marker of inflammation, in men and women with newly diagnosed Type 2 Diabetes mellitus (DM) in a population-based setting.

**Methods:**

CRP was measured in 1,037 patients (57% male) with newly diagnosed Type 2 DM included in the prospective nationwide Danish Centre for Strategic Research in Type 2 Diabetes (DD2) project. We assessed the prevalence of elevated CRP and calculated relative risks (RR) examining the association of CRP with lifestyle and clinical factors by Poisson regression, stratified by gender. We used linear regression to examine the association of CRP with other biomarkers.

**Results:**

The median CRP value was 2.1 mg/L (interquartile range, 1.0 – 4.8 mg/L). In total, 405 out of the 1,037 Type 2 DM patients (40%) had elevated CRP levels (>3.0 mg/L). More women (46%) than men (34%) had elevated CRP. Among women, a lower risk of elevated CRP was observed in patients receiving statins (adjusted RR (aRR) 0.7 (95% confidence interval (CI) 0.6-0.9)), whereas a higher risk was seen in patients with central obesity (aRR 2.3 (95% CI 1.0-5.3)). For men, CRP was primarily elevated among patients with no regular physical activity (aRR 1.5 (95% CI 1.1-1.9)), previous cardiovascular disease (aRR1.5 (95% CI 1.2-1.9) and other comorbidity. For both genders, elevated CRP was 1.4-fold increased in those with weight gain >30 kg since age 20 years. Sensitivity analyses showed consistent results with the full analysis. The linear regression analysis conveyed an association between high CRP and increased fasting blood glucose.

**Conclusions:**

Among newly diagnosed Type 2 DM patients, 40% had elevated CRP levels. Important modifiable risk factors for elevated CRP may vary by gender, and include low physical activity for men and central obesity and absence of statin use for women.

## Background

In patients with Type 2 diabetes mellitus (DM), the level of systemic inflammation, measured by C-reactive protein (CRP), may be a predictor of cardiovascular disease (CVD) and worse prognosis [1,2]. Thus, for prevention purposes, early detection of elevated CRP levels and identification of modifiable factors associated with this condition are important [3]. Information regarding elevated CRP levels and associated factors at the time of Type 2 DM diagnosis in a population-based setting is limited. In cross-sectional studies of the general population, elevated levels of CRP are often found in older people, in those with high Body Mass Index, and in less physically active people [4,5]. Lower CRP levels have been reported among individuals with regular alcohol consumption and those under statin treatment [5,6]. We thus aimed to examine the prevalence of and modifiable factors associated with elevated CRP among newly diagnosed Type 2 DM patients included in the nationwide Danish Centre for Strategic Research in Type 2 Diabetes (DD2) cohort study. As CRP is higher in females than males [7,8], we also wished to examine whether factors associated with elevated CRP in Type 2 DM differed between genders.

## Methods

We conducted this prevalence study using cross-sectional baseline data from DD2, a nationwide cohort study of newly diagnosed Type 2 DM patients enrolled from the practices of general practitioners (GPs) and hospital specialist outpatient clinics in Denmark since November 2010 [9]. At enrolment into the study cohort 66% were on antidiabetic treatment [10], among the patients included in this current study 71% started within one year prior to study start or after. The implementation and logistics of the DD2 project, patient enrollment, and the DD2 biobank have been described previously [11]. In brief, GPs or hospital physicians enter detailed interview and clinical examination data into the DD2 database (questionnaire provided in [11]). Blood (fasting) and urine samples are obtained from each patient, either on the day of the interview or during a later visit.

Linkage of data among different Danish medical and administrative registries is possible via a unique personal identifier (CPR number) provided to each resident at birth or upon immigration [12]. The CPR number allowed data linking of the DD2 cohort with other Danish registries.

### Lifestyle -, clinical factors and biomarkers

From the DD2 database, we extracted data on age, gender, high alcohol intake, regular physical activity, waist circumference (central obesity) and weight gain since age 20 years, as described in detail by Nielsen *et al.*[11].

The complete hospital contact history of each participant was obtained through linkage with the Danish National Registry of Patients (DNRP), which covers discharge records from all Danish hospitalizations since 1977 [13] and hospital outpatient visits since 1995. Diagnoses in the DNRP are coded according to the *International Classification of Diseases* (ICD), 8^th^ revision (ICD-8) codes until 1994 and 10^th^ revision (ICD-10) thereafter. From the DNRP, we obtained information on patients’ major chronic disease diagnoses since 1977, defined as those included in the Charlson Comorbidity Index (CCI) [14]. Based on hospital diagnosis codes (ICD-8 and ICD-10) for these conditions [15], we computed a CCI score for each person, excluding diabetes. We then defined three comorbidity levels: low (score of 0), medium (score of 1–2), and high (score of 3+). Diabetes was excluded from the CCI as it constituted the index disease of our study population. We separately ascertained previous diagnoses of any cardiovascular disease.

Complete data on antihypertensive and hypolipidemic treatment for each Type 2 DM patient were obtained through linkage with the Danish National Database of Reimbursed Prescriptions [16].

From the DD2 Biobank we also extracted information on the following biomarkers: Alanine Transferase (ALAT) levels, measured by the photometric method using the COBAS-6000 analyser, Roche Diagnostics; amylase levels, measured using an enzymatic colorimetric method (Pancreas-α-amylase); C-peptide levels, measured using the ADVIA Centaur C-Peptide assay (Siemens Healthcare Diagnostics Ltd, Frimley, Camberley, UK); and fasting blood glucose levels, analyzed using a enzymatic hexokinase method (Gluco-quant Glucose/HK, Roche Diagnostics).

### C-reactive protein (CRP)

From the DD2 biobank we extracted information on CRP levels, which were measured in the first 1,037 Type 2 DM patients who enrolled in the DD2 project. The particle-enhanced immunoturbidimetric method using Tina-quant C-reactive Protein Gen.3 (Roche Diagnostics GmbH, Mannheim, Germany), was used to measure CRP, with the possibility of measuring CRP within the limits of 0,3 - 350 mg/l; this is not a high-sensitivity CRP measurement. Elevated CRP levels was classified as CRP levels over >3.0 mg/L according to the guidelines of the Centers for Disease Control and the American Heart Association [17].

Patient registration and sample collection for the DD2 project have been approved by the National Committee on Health Research Ethics (Denmark) (record number S-20100082) and the Danish Data Protection Agency (record number 2008-58-0035). After receiving detailed oral and written information approved by the National Committee on Health Research Ethics (Denmark), patients volunteer to participate in the DD2 project and sign a written informed consent document.

### Statistical analysis

We calculated the median CRP value and examined the number of Type 2 DM patients within pre-specified groups defined in terms of demographic -, lifestyle-, and clinical characteristics. The prevalence of elevated CRP was calculated as the proportion of patients with a CRP value > 3 mg/L.

In order to exclude patients with underlying conditions such as infection, we also conducted two sub-analyses, one restricted to individuals with CRP levels ≤10 mg/L, the second restricting to individuals not hospitalized 14 days prior to entry into the study.

We calculated crude and adjusted relative risks (RR) of elevated CRP and their corresponding 95% confidence intervals (CI), comparing patients with and without different factors, stratified by gender. We used sequential cumulative adjustment models in the Poisson regression analyses with robust error variance, controlling first for age; then central obesity. In the full model we adjusted for age, central obesity, comorbidity level, physical activity, and high alcohol intake.

To examine the association of CRP levels with biomarkers, measured as continuous variables, we performed linear regression analysis. In all linear regression analyses, a normal distribution was approximated by log-transforming the variables. Multiple linear regression analysis was used. In the first step we adjusted for age and gender (Model 1); in the second step we adjusted for age, gender, and waist circumference (Model 2); and in the third model we adjusted for age, gender, waist circumference, ALAT, C-peptide, and fasting blood glucose levels.

All analyses were performed using SAS version 9.2 (SAS Institute, Inc., Cary, North Carolina).

## Results

In the 1,037 newly diagnosed Type 2 DM patients (43% women, 57% men), the distribution of CRP levels was skewed to the right, with a median value of 2.1 mg/L (interquartile range, 1.0 – 4.8 mg/L), ranging from 0 to 69.8 mg/L. In total, 405 out of 1,037 patients (40%) had elevated CRP levels (>3.0 mg/L), with more females (46%) than males (34%) having elevated CRP. A CRP level of 6 mg/L or more was observed in 20% of the patients, while 97 (9%) had a CRP level >10 mg/L.

Table 1 shows demographic, clinical, and lifestyle characteristics according to CRP levels stratified by gender, and the corresponding crude and fully adjusted relative risks, with 95% CI (data not shown for the adjusted model with age alone, and age + central obesity, as there were few differences between these models and the full model).

**Table 1:** Lifestyle and clinical factors of 1,037 newly diagnosed T2 DM patients in the Danish population-based DD2 project according to CRP levels and stratified on gender, with corresponding relative risks (RR) for elevated CRP

	**Females**				**Males**			
	**CRP levels <3.0 mg/L (n = 244)**	**CRP levels > 3.0 mg/L (n = 205)**	**Crude RR of CRP >3.0 mg/L (95% CI)**	**Adjusted RR**^**a **^**of CRP >3.0 mg/L (95% CI)**	**CRP levels <3.0 mg/L (n = 388)**	**CRP levels > 3.0 mg/L (n = 200)**	**Crude RR of CRP >3.0 mg/L (95% CI)**	**Adjusted RR**^**a **^**of CRP >3.0 mg/L (95% CI)**
	**N (%)**	**N (%)**			**N%**	**N%**		
**Age**								
Age < 40 years	8 (35)	15(65)	1.73 (1.23-2.43)	1.41 (0.97-2.04)	18 (59)	13 (41)	1.34 (0.89-2.17)	1.61 (1.03-2.52)
Age 40-59 years	84 (46)	98 (54)	1.43 (1.16-1.76)	1.35 (1.09-1.66)	145 (62)	90 (38)	1.26 (1.00-1.59)	1.30 (1.03-1.62)
Age 60+ years	152 (62)	92(38)	1	1	224 (70)	97 (30)	1	1
**Charlson comorbidity index score**								
0	172 (55)	139 (45)	1	1	283 (70)	119 (30)	1	1
1-2	57 (52)	54 (48)	1.10 (0.86-1.37)	1.15 (0.91-1.44)	92 (59)	63 (41)	1.37 (1.0-1.75)	1.47 (1.15-1.89)
3+	15 (56)	12 (44)	0.99 (0.64-1.54)	1.03 (0.66-1.60)	13 (42)	18 (58)	1.96 (1.40-2.74)	2.01 (1.44-2.98)
**Any previous cardiovascular disease**								
No	213 (54)	189 (46)	1	1	307 (69)	139 (31)	1	1
Yes	31 (51)	30 (49)	1.05 (0.79-1.38)	1.18 (0.90-1.56)	81 (57)	61 (43)	1.38 (1.09-1.74)	1.50 (1.17-1.91)
**High alcohol intake**^**b**^								
No	235 (54)	196 (46)	1	1	347 (65)	183 (35)	1	1
Yes	9 (50)	9 (50)	1.10 (0.68-1.77)	1.20 (0.77-1.87)	41 (70)	17 (30)	0.85 (0.56-1.28)	0.88 (0.59-1.32)
**Physical activity**								
Regular	118 (59)	84 (41)	1	1	146 (75)	48 (25)	1	1
None	126 (51)	121(49)	1.18 (0.96-1.45)	1.14 (0.93-1.40)	242 (61)	142 (39)	1.49 (1.13-1.97)	1.46 (1.11-1.91)
**Central obesity**^**c**^								
No	22 (81)	5 (19)	1	1	40 (77)	12 (23)	1	1
Yes	222 (53)	200 (47)	2.55 (1.15-5.68)	2.27 (1.00-5.16)	347 (65)	188 (35)	1.52 (0.91-2.53)	1.42 (0.83-2.41)
**Weight gain >30 kg since 20 years of age**								
No	164 (61)	103 (39)	1	1	256 (72)	100 (28)	1	1
Yes	80 (44)	102 (56)	1.45 (1.19-1.77)	1.36 (1.11-1.66)	132 (57)	100 (43)	1.54 (1.23-1.92)	1.42 (1.13-1.78)
**Antihypertensive treatment**								
No	75 (67)	56 (43)	1	1	126 (70)	52 (30)	1	1
Yes	169 (21)	149(47)	1.10 (0.87-1.38)	1.23 (0.97-1.57)	162 (64)	148 (36)	1.63 (1.26-2.11)	1.24 (0.95-1.62)
**Statin treatment**								
No	61 (43)	83 (57)	1	1	124 (64)	66 (35)	1	1
Yes	183 (60)	122 (40)	0.69 (0.57-0.84)	0.75 (0.61-0.91)	264 (66)	134 (34)	0.96 (0.76-1.23)	0.96 (0.41-1.89)

^a^Adjusted for age, gender, central obesity, comorbidity, physical activity and alcohol consumption (except when stratified by given variable).

^b^High alcohol intake defined as (≥14/21 alcoholic drinks/week for women/men).

^c^Central obesity defined as waist circumference ≥94 cm men/≥80 cm women.

For female patients, a lower risk of elevated CRP was observed in patients receiving statins (adjusted relative risk (aRR) 0.7 (95% confidence interval (CI) 0.6-0.9)) compared to patients not receiving statins, while a substantially higher risk was seen in patients with central obesity (aRR 2.3 (95% CI 1.0-5.3)) compared to patients without central obesity (Table 1).

For males, a higher risk of elevated CRP was observed in particular in patients with no regular physical activity (aRR 1.5 (95% CI 1.1-1.9)) compared to males with regular physical activity, and in those with previous cardiovascular disease (aRR 1.5 (95% CI 1.2-1.9) compared to no cardiovascular disease. Additionally, higher risk was observed in male patients with a moderate or high overall CCI score (aRR 1.5 (95% CI 1.2-1.9) for scores of 1-2 and 2.07 (95% CI 1.4-2.9) for scores of 3+) compared to patients with a score of 0. Increased risk of elevated CRP was also seen among males with central obesity (although to a smaller extent than in females), and decreased risk was associated with higher alcohol intake, but statistical precision was limited for these estimates.

For both genders, a higher risk was observed among patients with weight gain >30 kg since age 20 years (for women aRR 1.4 (95% CI 1.1-1.79); for men aRR 1.4 (95% CI 1.1-1.8)), and among patients younger than 60 years (for women, <40 years aRR 1.4 (95% CI 1.0-2.0) and 40-69 years aRR 1.4 (95% CI 1.1-1.7); for men, <40 years aRR 1.6 (95% CI 1.0-2.5) and 40-69 years aRR 1.3 (95% CI 1.0-1.6)) compared to over 60 years.

The sub-analyses, restricting the analysis on patients with a CRP level of ≤10 mg/L and the analyses restricting on no previous hospitalization the past 14-days showed consistent results with the full analysis.

In the linear regression analysis, (Table 2), increasing fasting blood glucose (adjusted β (Model 3) 0.03 (95% CI 0.006- 0.04)) showed a weak but positive association with elevated CRP, after adjusting for age, gender, waist circumference, and other biomarker levels.

**Table 2:** Regression analysis of biomarkers and the association with CRP levels in 1037 patients with newly diagnosed Type 2 DM patients

	**B-coeff Crude (95%CI)**	**Stand. Beta Crude (95%CI)**	**B-coeff Model 1**^**a **^**(95%CI)**	**Stand. Beta Model 1**^**a **^**(95%CI)**	**B-coeff Model 2**^**b **^**(95%CI)**	**Stand. Beta Model 2**^**b **^**(95%CI)**	**B-coeff. Model 3**^**c **^**(95%CI)**	**Stand. Beta Model 3**^**c **^**(95%CI)**
ALAT^d^	0.025	0.06	0.024	0.05	-0.003	-0.009	-0.01	-0.03
	(-0.003,0.05)	(-0.0006,0.12)	(-0.002,0.05)	(-0.005,0.11)	(-0.03,0.02)	(-0.07,0.05)	(-0.05, 0.04)	(-0.13,0.07)
Amylase^d^	-0.019	-0.04	-0.013	-0.03	-0.001	-0.002	0.03	0.06
	(-0.048,0.01)	(-0.01,-0.06)	(-0.04,0.017)	(-0.09,0.04)	(-0.33, 0.03)	(-0.07,0.06)	(-0.01, 0.08)	(-0.04,0.17)
C-peptide^d^	0.12	0.29	0.12	0.29	0.05	0.0004	0.02	0.07
	(0.09-0.14)	(0.23,0.35)	(0.098,0.14)	(0.23-0.35)	(0.03,0.07)	(-0.05,0.05)	(-0.01, 0.06)	(-0.02,0.16)
Fasting blood glucose^d^	0.03	0.19	0.03	0.2	0.03	0.15	0.03	0.14
	(0.02,0.04)	(0.13,0.24)	(0.02,0.04)	(0.11,0.23)	(0.015-0.04)	(0.08,0.21)	(0.006, 0.04)	(0.03,0.25)

^a^Adjusted for age and gender.

^b^Adjusted for age, gender, central obesity.

^c^Adjusted for age, gender central obesity, all biomarkers (except when stratified by given variable).

^d^All biomarkers (including CRP) are log transformed.

## Discussion

This study, using cross-sectional baseline data from the nationwide DD2 cohort and biobank, show that about 40% of newly diagnosed Type 2 DM patients in Denmark have an elevated CRP level (more than 3 mg/L). Several potentially modifiable factors, such as lack of physical activity, weight gain, central obesity, absence of statin treatment, and high fasting blood glucose are associated with elevated CRP levels in Type 2 DM, but the strengths of these associations may vary by gender.

The results from our explorative study provide new knowledge about CRP levels and associated factors in newly diagnosed Type 2 diabetes patients as compared with previous cross-sectional studies of the general population or persons with prevalent Type 2 DM [5,8,18]. In line with previous evidence in the general population, we found that central obesity independently is associated with CRP elevation at Type 2 DM debut [5], but primarily for females. This is in line with previous knowledge that the contributing effect of adiposity to elevated CRP levels may be especially relevant for women compared to men [19]. The causal pathways and time sequence between elevated CRP levels and central obesity, insulin resistance, and the metabolic syndrome are complicated and remain largely unresolved.

We also found that statin treatment was associated with lower risk of elevated CRP levels, consistent with previous findings from the general population [5]; possibly due to the anti-inflammatory effects of statins. Interestingly, this was only seen for females in our study. The explanation remains unclear as recent meta-analyses report that statins work similarly between the genders [20]. The present results suggest that an anti-inflammatory effect of statins may be expected particular among women.

However, there are also some discrepancies between our study and previous literature. In contrast with observations in the general population we found that in newly diagnosed type 2 DM patients, young age at DM debut was associated with substantially higher prevalence of CRP than older ages, after controlling for differences in central obesity and other factors. It is thus possible that a high degree of systemic inflammation and a tendency towards early Type 2 DM debut travel together.

The 40% prevalence rate of elevated CRP found in our population-based sample of incident Type 2 DM patients in Denmark is similar to levels of elevated CRP found among Hispanics and African-Americans with incident Type 2 DM in the prospective Multi-Ethnic Study of Atherosclerosis (40%) [21]. The prevalence is slightly higher than in previously reported cross-sectional samples of white/European adults from the general population; ranging from 28%-35% [4,5,22] in the adult Spanish population and the general US population included in the 1999-2000 National Health and Nutrition Examination Survey.

Elevated CRP has been associated with increased incidence of subsequent cardiovascular events and cardiovascular mortality in Type 2 DM patients [2,23]. For example, in the WOSCOP study, patients with the metabolic syndrome and elevated CRP had increased cardiovascular mortality (RR 2.75 (95% CI, 2.1-3.6)) compared to patients without the metabolic syndrome and with low CRP values. While the clinical usefulness of CRP measurement and causality relative to cardiovascular events has been questioned [24,25], CPR measurements may help to identify subgroups of Type 2 DM patients at higher risk for comorbid disease. Of note, we observed that high CRP level was associated with substantially increased overall comorbidity and with previous cardiovascular disease mainly among men at time of Type 2 DM debut. It would also be of interest to examine the relationship of diabetic microvascular complications, such as diabetic neuropathy, with CRP elevation, but reliable information on microvascular complications was unfortunately not available in our present data. CRP measurements may also identify Type 2 diabetes patients for whom treatment of cardiovascular risk factors or diabetes may be particularly beneficial. Vepsalainen and colleagues found that physical activity reduced cardiovascular events and mortality only for Type 2 DM patients with CRP levels above 3 mg/L [26]. For a subgroup of patients with CRP levels higher than the median, Strom and colleagues showed that atorvastatin may slow the decline of beta cell function in patients with type 1 DM [27].

The main strength of our study is its comprehensive and detailed assessment of lifestyle, clinical, and biomarkers based on the DD2 database and biobank, with close to 100% completeness for these variables. Additionally, linkage with the DNRP provided detailed clinical information on patients with the Type 2 DM. Study limitations include its cross-sectional design, leading to uncertainty about whether increased inflammation and elevated CRP levels precede or follow clinical and metabolic changes. In addition, as discussed by Thomsen *et al.,* the DD2 cohort likely represents patients whose newly diagnosed Type 2 DM is more severe than average in Denmark, as enrolment still relies much on hospital outpatient clinics [10]. We only have information of co-morbidities registered in DNRP, requiring in- or out-patient hospital care. Thus, chronic diseases, taking long time to develop and not requiring specialized care in the early phases, would not be captured. The assay, albeit not being a hs-CRP measurement, conveys the same sensitivity as a hs-CPR measurement.

## Conclusions

T2DM patients with elevated CRP are likely to benefit from targeted gender-specific lifestyle interventions [26,28], for females including weight loss and potentially statin treatment, while for males, physical activity seems particularly important. Future prospective follow-up studies of the DD2 study cohort will increase our understanding of how CRP elevation is associated with the clinical course of Type 2 DM.

## Competing interests

The authors declare that they have no competing interests.

## Authors’ contributions

JSC, HBN, HTS and JR participated in designing the DD2 cohort. JR, JSN, SF, IB, JSC, HBN, HTS and RWT conceived of the study. IB was responsible for the biochemical analyses. ES, AM, RWT and HTS participated in the design of the study and KB performed the statistical analysis. ES initially drafted the article, with help by RWT and HTS. All other authors have critically reviewed the manuscript. All authors read and approved the final manuscript.

## Pre-publication history

The pre-publication history for this paper can be accessed here:

http://www.biomedcentral.com/1472-6823/14/74/prepub
